# Correction: Metabolite changes during developmental transitions in *Adonis amurensis* Regel et Radde flowers: Insights from HPLC-MS analysis

**DOI:** 10.1371/journal.pone.0321792

**Published:** 2025-04-02

**Authors:** Zhu Xingzun, Wang Hongtao

The first author’s name is spelled incorrectly. The correct name is: Zhu Xingzun.

The following information is missing from the Funding statement: Natural Science Foundation of Jilin Province (YDZJ202501ZYTS575).
