# Supplementary material for: Metabolite changes during developmental transitions in Adonis amurensis Regel et Radde flowers: Insights from HPLC-MS analysis
Source: PLoS One. 2025 Jan 6;20(1):e0313337. doi: 10.1371/journal.pone.0313337 (PMC11703111; doi:10.1371/journal.pone.0313337)
Supplement: Attachment — Submitted filename: Response to Reviewers.docx [file pone.0313337.s003.docx]

**Response to Reviewer's Questions**

**Comments to the Author**

1. Is the manuscript technically sound, and do the data support the conclusions?

The manuscript must describe a technically sound piece of scientific research with data that supports the conclusions. Experiments must have been conducted rigorously, with appropriate controls, replication, and sample sizes. The conclusions must be drawn appropriately based on the data presented.

Reviewer #1: No

Reviewer #2: Yes

2. Has the statistical analysis been performed appropriately and rigorously?

Reviewer #1: No

Reviewer #2: Yes

3. Have the authors made all data underlying the findings in their manuscript fully available?

The [PLOS Data policy](http://www.plosone.org/static/policies.action#sharing) requires authors to make all data underlying the findings described in their manuscript fully available without restriction, with rare exception (please refer to the Data Availability Statement in the manuscript PDF file). The data should be provided as part of the manuscript or its supporting information or deposited to a public repository. For example, in addition to summary statistics, the data points behind means, medians and variance measures should be available. If there are restrictions on publicly sharing data—e.g. participant privacy or use of data from a third party—those must be specified.

Reviewer #1: No

Reviewer #2: Yes

4. Is the manuscript presented in an intelligible fashion and written in standard English?

PLOS ONE does not copyedit accepted manuscripts, so the language in submitted articles must be clear, correct, and unambiguous. Any typographical or grammatical errors should be corrected at revision, so please note any specific errors here.

Reviewer #1: No

Reviewer #2: Yes

5. Review Comments to the Author

**Reviewer #1: Dear authors**
Adonis amurensis Regel et Radde holds significant importance as a spring ephemeral
plant. It’s medicinal uses have been extensively studied in targeting cardiac insufficiency and edema. Its anticancer potential has also been identified. Previous investigation delineated its physical characteristics, flowering patterns, reproductive and cultural biology and molecular biology. However, the flower developmental stage associated metabolic changes are poorly understood. This study aimed to investigate the changes in metabolites and their interactions at five developmental stages, namely Flower Primordium (FP), Sepal Stage (SE), Perianth Primordium, (PE) Stamens Stage (SE), and Pistil Stage (PI). Hight performance liquid chromatography coupled with mass spectroscopy was performed to identify the flower developmental stage associated metabolites. The analysis of the data was done through MSEA and KEGG database was employed for metabolite annotation. Flower development stages significantly impacted both the quantity and quality of metabolite contents, with notable shifts in the types and amounts of metabolites occurring throughout the flower development process. Particularly, the SE vs PI stage exhibited the highest number (453) of significantly differentiated metabolites, indicating a substantial increase in metabolite expression during the development of reproductive organs. Conversely, the PE vs ST stage showed the lowest number of significantly differentiated metabolites.

The study provides novel insights into the analysis of distinct metabolite contents at
each stage of floral development. The information obtained will facilitate better
understanding of studying the factors involved in flower development and transition
from one stage to another.

Based on the provided abstract and supporting materials. I couldn't access the main draft due to the authors not having uploaded the full text of the article; therefore, I am making my decision as a rejection.

Authors’ response: We are sorry this reviewer could not access our manuscript draft. We uploaded the manuscript draft to the online system and the supporting materials.

Reviewer #2: The paper entitled Metabolimics revealed a comprehensive understanding of the biochemical and genetic nechanisms underlying the developmental shift of the Adonis amurensis Regel et Raddle flowers pertaining to metabolic analysis at differnet stages of floral development. It has been previously reported in number of plant species including the model plants that there is differential expression of genes during developmental stages which transcribes into differential metabolic profiles.

The idea of the work is good and novel and pertains to identification of primary and secondary metabolites during floral development.

However, it would have been beneficial to mine unique compounds from the secondary metabolite groups identified in the study. This would help in future for collecting the plant material at the right stage for the bioactive/therapeutic compounds.

Authors’ response: We are thankful to the reviewer for his nice comment. We have provided this information for each group in sub-sections of the manuscript.

The language of the paper is comprehensive with few gramatical errors.

Authors' response: We have availed the services of a skilled person to revise our manuscript thoroughly.

The introduction and the discussion has repetitions which should be avaoided.

Authors' response: We have deleted the repetition parts.

I will suggest some changes here which could improve the paper for wider audience.

My suggestion is that the title should be changed since the biochemical and genetic work has not been performed (i.e, proteomics + transcriptomics) has not been done into this proper.

Authors response: We are thankful to the reviewer for his nice comment. We have revised the title of the manuscript.
It would be desirable if the photographs depicting FP, SE, PE, Se and Pi stages are incorporated in the proper way. This would give visual comprehension to the readers.

Authors' response: We have provided the Scanning electronic microscopic (SEM) images of *A. amurensis* developing flower at: flower primordium (FP), sepal stage (SE), perianth primordium (PE), stamen (ST), and pistil (PI) differentiation stages in Figure 1.

In Table 1 don’t use the words transcript (instead use replicates)
Kindly mention if the replicates were from the same plant or from the different plant

Authors' response: We have modified it accordingly.

Were the plants cultivated on the station or collected from wild

Authors’ response: The plants were collected from wild.

Use superscript for the abbreviation of ions.

Authors' response: We have modified accordingly.

In Discussion first ten lines are in repetition from introduction.

Authors' response: The repetition is avoided in the revised manuscript.

6. PLOS authors have the option to publish the peer review history of their article ([what does this mean?](https://journals.plos.org/plosone/s/editorial-and-peer-review-process#loc-peer-review-history)). If published, this will include your full peer review and any attached files.


If you choose “no”, your identity will remain anonymous but your review may still be made public.


**Do you want your identity to be public for this peer review?** For information about this choice, including consent withdrawal, please see our [Privacy Policy](https://www.plos.org/privacy-policy).

Reviewer #1: No

Reviewer #2: No
