# Supplementary material for: Metabolite changes during developmental transitions in Adonis amurensis Regel et Radde flowers: Insights from HPLC-MS analysis
Source: PLoS One. 2025 Jan 6;20(1):e0313337. doi: 10.1371/journal.pone.0313337 (PMC11703111; doi:10.1371/journal.pone.0313337)
Supplement: Attachment — Submitted filename: Comments about PONE-D-23-24707R1.docx [file pone.0313337.s004.docx]

Adonis amurensis Regel et Radde is a spring ephemeral plant with high ornamental value and can be used as medicine. The authors measured the changes in metabolites of *A. amurensis* flowers at various developmental stages, providing certain insights into the understanding of their development. Overall, this is a very meaningful study.

Although the author has made modifications according to the suggestions of reviewer 2, there are still some problems.

1 The scientific name of the species should be written completely when it appears for the first time in the manuscript. It can be abbreviated when it appears again later. Regarding the species *Adonis amurensis* Regel et Radde in this manuscript, it can be written as *A. amurensis* when it appears again later.

2 I don’t know whether it is a requirement of the journal or an oversight by the author, but generally manuscripts should have line numbers to facilitate review by reviewers.

3 Regarding the content of differential metabolite screening in the main text of the manuscript, I think the description is too complicated. The author can select the groups with more differential expressions to focus on them, and briefly introduce the rest. Relevant charts for groups with less differential expression can be placed in the attachment.

4 The first sentence in the discussion section is very strange. It should be a mistake in punctuation. Please check it carefully.

5 The description of differentially expressed metabolites in the Discussion section is too simplistic, and the authors should carefully review the literature and conduct a more in-depth discussion. In addition, the author described in the manuscript that ST and PI are significantly different compared with other stages, but what effect this difference has on the development of *A. amurensis* flowers, please discuss in depth.

6 The author studied the changes in metabolites of *A. amurensis* flowers at various developmental stages, but did not emphasize the specific significance of this study. The author has repeatedly emphasized that *A. amurensis* is a medicinal plant and can treat a variety of diseases. So what significance does this research have for the resource utilization of *A. amurensis*, especially in the treatment of diseases? Authors should describe relevant content in the Discussion and briefly mention it in the Abstract.

7 There is too much description of background and methods in the abstract, and the results should be added with specific differentially expressed metabolites.
