# Supplementary material for: Metabolite changes during developmental transitions in Adonis amurensis Regel et Radde flowers: Insights from HPLC-MS analysis
Source: PLoS One. 2025 Jan 6;20(1):e0313337. doi: 10.1371/journal.pone.0313337 (PMC11703111; doi:10.1371/journal.pone.0313337)
Supplement: Attachment — Submitted filename: R2_Response to reviewers PONE-D-23-24707R1.docx [file pone.0313337.s005.docx]

**Response to the reviewer’s comments**

We would like to express our sincere gratitude to the reviewers for the thorough and insightful comments provided on our manuscript. We greatly appreciate the time and effort invested in evaluating our work.

We have carefully considered and addressed all the comments and suggestions provided by the reviewers. Their valuable feedback has significantly enhanced the scientific readability and overall quality of our paper.

Below is a detailed response to each of the reviewers' comments, outlining the changes made to the manuscript in response to their suggestions. We believe that these revisions have strengthened our study and improved the clarity and impact of our findings.

We are confident that these revisions will be helpful to satisfy the reviewers questions, but if there is still need of any further improvement, we are more than happy to follow the reviewers’ instructions.

**Reviewer’s comments and responses**

Adonis amurensis Regel et Radde is a spring ephemeral plant with high ornamental value and can be used as medicine. The authors measured the changes in metabolites of *A. amurensis* flowers at various developmental stages, providing certain insights into the understanding of their development. Overall, this is a very meaningful study.

Although the author has made modifications according to the suggestions of reviewer 2, there are still some problems.

1. The scientific name of the species should be written completely when it appears for the first time in the manuscript. It can be abbreviated when it appears again later. Regarding the species *Adonis amurensis* Regel et Radde in this manuscript, it can be written as *A. amurensis* when it appears again later.

Author’s response: We are thankful to the reviewer for his constructive feedback regarding the presentation of scientific names in our manuscript. We have carefully reviewed the whole manuscript to correct usage of the species name "*Adonis amurensis* Regel et Radde." In response, we have revised the manuscript to ensure that the full scientific name of the species is used upon its first mention. For subsequent references, we have abbreviated the name to "*A. amurensis*" in accordance with standard scientific writing practices. This change has been applied throughout the document to maintain consistency and adhere to the correct format for scientific names.

2. I don’t know whether it is a requirement of the journal or an oversight by the author, but generally manuscripts should have line numbers to facilitate review by reviewers.

Author’s response: We have added the line numbers in the revised version of the manuscript.

3. Regarding the content of differential metabolite screening in the main text of the manuscript, I think the description is too complicated. The author can select the groups with more differential expressions to focus on them, and briefly introduce the rest. Relevant charts for groups with less differential expression can be placed in the attachment.

Author’s response: We are thankful to the reviewer for his deep insights regarding the section on differential metabolite screening in our manuscript. We have carefully considered your suggestion to simplify the description by focusing on groups with more significant differential expressions and briefly introducing the rest, with additional charts for groups with less differential expression placed in the supplementary files.

After a thorough review of the manuscript and the supplementary materials already provided, we have determined that the current level of detail and the structure of the presentation are essential to provide detailed description of differential metabolite screening to the readers to fully grasp the scope and significance of our findings. It ensures that the methodology and results are transparent and reproducible. Moreover, the groups with less differential expression, although seemingly minor, play a critical role in the context of our study's broader findings. They contribute to the overall understanding of the metabolic pathways involved and cannot be adequately represented solely through supplementary materials. In addition, we have ensured that the supplementary files provided with the manuscript already include extensive supportive data and visualizations that complement the main text. These materials have been organized to enhance the reader's ability to understand deeper the areas of specific interest without overcomplicating the main text.

In light of these considerations, we have opted to maintain the current structure and depth of the differential metabolite screening description in the manuscript. We believe this approach best serves the scientific community's interest by providing a detailed and comprehensive presentation of our research findings.

We appreciate your understanding and value your suggestions, which have prompted a careful review of our manuscript. It is assured that we have revisited both the main text and supplementary materials to ensure clarity and accessibility of the information presented.

4. The first sentence in the discussion section is very strange. It should be a mistake in punctuation. Please check it carefully.

Author’s response: We are thankful to the reviewer for his thorough review and for pointing out the concerns regarding the complexity and punctuation of the opening sentence in the “Discussion” section of our manuscript. We appreciate the reviewer’s attention to this mistake. In response, we have carefully revised the first sentence of the “Discussion” section.

5. The description of differentially expressed metabolites in the “Discussion” section is too simplistic, and the authors should carefully review the literature and conduct a more in-depth discussion. In addition, the author described in the manuscript that ST and PI are significantly different compared with other stages, but what effect this difference has on the development of *A. amurensis* flowers, please discuss in depth.

Author’s response: We are thankful to the reviewer for his/her constructive comments and the opportunity to enhance our manuscript. We acknowledge the need for a more detailed discussion regarding the differentially expressed metabolites and their implications in the development of *Adonis amurensis* flowers, specifically during the ST (Stamens Stage) and PI (Pistil Stage). In our revised version of the manuscript, the “Discussion” section is supported by the newer information regarding the implications of the differentially expressed metabolites identified between the ST and PI stages. These stages represent critical transitions in floral development, where metabolomic changes are closely linked to the differentiation of male and female reproductive structures, respectively.

In our findings, we have observed significant metabolic shifts involving pathways such as flavonoid biosynthesis, flavone and flavonol biosynthesis. In addition, there was a significant shift in plant hormone signal transduction. These pathways are involved in flower coloration, growth regulation, and the development of reproductive organs. The flavonoid biosynthesis pathway not only contributes to the pigmentation and visual attraction of the flowers but also provides UV protection and aids in fertility. Studies such as those by Zhao et al. [1] and others [2, 3] have demonstrated the critical functions of flavonoids in flower development and pollinator attraction. The significant differences in metabolite expression between the ST and PI stages underscore the distinct metabolic demands of these developmental phases, the upregulation of metabolites in the PI stage, especially those involved in the biosynthesis of secondary metabolites and plant hormones, highlights the metabolic intensification important for ovule development and ultimately the formation of seed. This phenomenon is strongly supported by findings from Önder et al. [4], who noted the role of secondary metabolites in flower development in *Rosa damascena*. Moreover, the differential expression of metabolites involved in amino acid biosynthesis pathways, like the valine, leucine, and isoleucine biosynthesis pathways detected in our research, suggests an enhanced protein synthesis activity in the pistil to support ovary development and seed maturation. Similar findings were reported by Chakraborty et al. [5], emphasizing the role of amino acids in floral organ development. These findings are strongly supported by the literature [6-8], providing a comparative analysis that highlights how *A. amurensis* adapts its metabolic pathways to environmental cues and developmental needs. The incorporation of metabolomic data with previously reported transcriptomic and metabolomic data offers a complete view of the regulatory mechanisms in flower development.

6. The author studied the changes in metabolites of *A. amurensis* flowers at various developmental stages, but did not emphasize the specific significance of this study. The author has repeatedly emphasized that *A. amurensis* is a medicinal plant and can treat a variety of diseases. So what significance does this research have for the resource utilization of *A. amurensis*, especially in the treatment of diseases? Authors should describe relevant content in the Discussion and briefly mention it in the Abstract.

Author’s response: We are thankful to the reviewer for his/her insightful comment. We acknowledge the importance of clarity regarding the relevance of our findings to the medicinal utility of *A. amurensis*, particularly how these findings could impact its resource utilization for therapeutic purposes. We have updated the Discussion section and the Abstract to better highlight these implications. In the revised Abstract, we have added the following newer information. Our research not only helps us in understanding the metabolomic dynamics during the flower development of *A. amurensis* but also emphasizes the potential pharmacological implication of stage-specific metabolites. Identifying these metabolites can help targeted bioprospecting and optimization of extraction methods to tackle the plant’s full therapeutic potential, particularly in the development of treatments for cardiac insufficiency, edema, and possibly cancer. In the Discussion, we have expanded our explanation of the practical implications of our research findings in the context of medicinal use. We illustrate how the stage-specific identification of metabolites can lead to more efficient use of plant resources, with potential cost and environmental benefits: The significant variations in the concentration and presence of key metabolites found in our study, such as flavonoids and saponins, across different developmental stages of *A. amurensis* flowers; are known for their anti-inflammatory, antioxidant, and cardio-protective properties [1-3]. By finding the stages at which these metabolites are most rich, our study advocates best harvest times that could improve the efficacy and yield of medicinal extracts derived from this plant. For example, if specific anti-cancer or cardio-protective metabolites peak during the Pistil stage, as suggested by our analysis, targeted harvesting during this stage could boost the medical efficacy of extracts used in treatment formulations. This approach is supported by the findings from other pharmacological studies, which have shown the therapeutic consequence of timing in plant metabolite extraction [4]. Understanding the dynamic changes in metabolite profiles during flower development stipulates crucial insights for agricultural practices and the sustainable use of *A. amurensis* as a medicinal resource. This knowledge can model cultivation strategies to enhance yield and quality, while also informing conservation efforts, especially given the plant’s status as a valuable but potentially vulnerable medicinal species. Moreover, our research can encourage advancements in phytopharmacology and herbal medicine by detecting candidate compounds for further investigation in drug development processes.

7. There is too much description of background and methods in the abstract, and the results should be added with specific differentially expressed metabolites.

Author’s response: Thank you for your valuable feedback regarding the composition of our abstract. We understand the importance of concisely conveying the main findings of our research, including specific results on differentially expressed metabolites. We have revised the abstract to streamline the background and methods information and emphasize the key results more explicitly.

Reviewer #1: Dear Authors,

Insights from HPLC-MS Analysis; Regrettably, upon the initial submission of the article, I encountered technical difficulties and was unable to access the files for examination.
I am pleased to inform you that I have now successfully accessed and thoroughly reviewed the manuscript. I appreciate the depth of research and the valuable insights presented in the study. The comprehensive analysis of metabolite changes during developmental transitions in *Adonis amurensis* flowers using HPLC-MS is commendable.
Throughout my evaluation, I observed the rigorous methodology employed and the significance of the findings in contributing to our understanding of metabolic dynamics during floral development. The use of HPLC-MS analysis has undoubtedly enriched the study and provided valuable data.
However, to enhance the overall quality of the manuscript, I would recommend specific recommendations and constructive. These suggestions are intended to contribute to the clarity, coherence, and impact of the article.
Once again, I appreciate the opportunity to engage with this research, and I am confident that addressing these points will further strengthen the manuscript.

1. Abstract

The abstract provides a comprehensive overview of the study on *Adonis amurensis* Regel et Radde, emphasizing its medicinal properties and previous research conducted on various aspects of the plant. However, there are several deficiencies in the abstract. Firstly, it lacks clarity in presenting the specific metabolic changes associated with each of the five developmental stages of the flower. The abstract mentions the use of HPLC-MS for metabolite characterization but does not provide any specific findings or identified metabolites. Additionally, there is a lack of detail on the methodologies employed in Metabolite Set Enrichment Analysis (MSEA) and the metabolite annotation process using the KEGG database. The abstract could be strengthened by including key findings or trends observed in metabolite changes across the developmental stages. Furthermore, it does not highlight the novelty or significance of the study in comparison to existing literature on *Adonis amurensis*. A brief mention of the potential implications of the findings for the plant medicinal properties or applications in the field of medicine and agriculture would enhance the abstract impact. Overall, while the abstract outlines the study objectives and methods, it falls short in providing specific results and contextualizing the significance of the research within the broader scientific landscape.

Author’s response: We are thankful to the reviewer for his/her constructive feedback regarding the abstract of our manuscript. We appreciate the opportunity to enhance the clarity and detail of our abstract to better highlight the specific results and their significance. In response to the comments, we have revised the abstract to incorporate specific metabolites identified, methodological details, and the broader implications of our findings.

2. Introduction

The introduction provides a comprehensive background on *Adonis amurensis*, covering its taxonomy, distribution, medicinal applications, and previous research areas. However, there are a few deficiencies in the introduction. Firstly, while the medicinal importance of *Adonis amurensis* is highlighted, the introduction lacks a clear statement of the gap or knowledge deficiency that the current study aims to address. It briefly mentions the previous studies on various aspects of *A. amurensis* but does not explicitly state the specific gap in understanding that necessitates the investigation of metabolic changes during flower development. Secondly, the transition between the general information on *Adonis amurensis* and the focus on flower development is abrupt. The introduction could benefit from a smoother transition that clearly outlines why studying flower development, particularly the metabolic aspects, is essential for addressing the knowledge gap. Furthermore, the paragraph discussing the importance of flowering in the life cycle of plants is somewhat general, and the link to the specific case of *Adonis amurensis* under extreme environmental conditions could be more explicitly articulated. The introduction would benefit from a stronger connection between the unique flowering characteristics of *A. amurensis* and the need for understanding the genetic regulatory mechanisms governing flower development under extremely low temperatures. Lastly, while metabolomics is introduced as a valuable approach, the specific relevance of metabolomic analysis to address the identified knowledge gap in *A. amurensis* flower development is not explicitly stated. The introduction could benefit from a more focused explanation of why metabolomics is particularly suited to unravel the genetic pathways and regulatory networks involved in flowering under challenging environmental conditions. In summary, the introduction provides a comprehensive background but could be strengthened by explicitly stating the knowledge gap, improving the transition between general information and the study focus, reinforcing the link between *A. amurensis* unique flowering characteristics and the research question, and clarifying the specific relevance of metabolomics in addressing the identified gap.

Author’s response: We are thankful to the reviewer for his/her thorough review and valuable feedback on the introduction part of our manuscript. We have revised the introduction to address the identified deficiencies. Specifically, we have explicitly stated the knowledge gap regarding the metabolic changes during flower development of *A. amurensis* under extreme environmental conditions. We improved the transition from general information to the study focus, providing a clear explanation of the importance of investigating flower development and its metabolic aspects. Additionally, we emphasized the unique flowering characteristics of *A. amurensis* and their relevance to understanding genetic regulatory mechanisms under low temperatures. We also clarified the relevance of metabolomics in uncovering genetic pathways and regulatory networks involved in flowering under challenging environmental conditions. These revisions ensure the introduction is comprehensive, cohesive, and directly addresses the reviewer's points.

3. Materials and Methods

Regarding the Materials and Methods section, there are some deficiencies in the section. Firstly, there is a lack of clarity in the description of the plant material collection process. The section mentions that transcripts were collected at different developmental stages, but it does not provide details on how the samples were collected, processed, and stored. Additionally, the rationale for selecting the specific developmental stages is not clearly articulated, and it would be beneficial to explain why these particular stages were chosen for analysis. Secondly, while the instrumentation systems and conditions for high-performance liquid chromatography/mass spectrometry (HPLC-MS) are mentioned, there is a lack of information on the sample preparation procedure prior to analysis. Details such as extraction methods, sample derivatization (if applicable), and any pre-processing steps are crucial for the reproducibility and reliability of the results, and their omission in the methods section is a deficiency. Furthermore, the section on qualitative and quantitative analysis of metabolites lacks specificity. Although the MWDB (Metware database) is mentioned, it would be helpful to provide more information on the database, including its content, reliability, and relevance to the study. Additionally, the description of the quality control (QC) samples and their role in ensuring reproducibility is brief, and it would benefit from more explicit details on how QC samples were prepared, monitored, and their impact on data quality assessed. The section on statistical analysis provides an overview of the software and methods used, but it lacks detail on the rationale behind choosing specific statistical approaches. Providing a brief explanation of why OPLS-DA and SPSS 20.0 were selected for data analysis would enhance the transparency and understanding of the statistical procedures.
In summary, the Materials and Methods section could be improved by providing more details on plant material collection, sample preparation, and the rationale for choosing specific developmental stages. Additionally, more specificity on the Metware database, QC sample handling, and the rationale behind statistical choices would enhance the rigor and clarity of the experimental methodology.

Author’s response: We are thankful to the reviewer for his/her constructive feedback on our manuscript. We have revised the Materials and Methods section to address the identified deficiencies. Specifically, we have added detailed information on the plant material collection process and sample preparation. This includes a clear description of how the samples were collected, processed, and stored at different developmental stages. In addition, we have added information regarding the identification of each distinct stage.

Additionally, we have provided more information on the MWDB (Metware database), including its content, reliability, and relevance to our study. The MWDB is a comprehensive metabolomics database that contains extensive metabolite information, ensuring high reliability and relevance for accurate metabolite identification and annotation in our analysis. Besides, we have added detailed information on the quality control and the statistical analysis. The revised section now ensures that all steps are thoroughly documented, providing the necessary clarity and completeness.

4. Results
   While your comprehensive analysis provides valuable insights into the metabolomic changes during the different stages of *Adonis amurensis* flower development, there are a few potential deficiencies that could be addressed to strengthen the scientific rigor of the study. Firstly, it would be beneficial to include statistical measures such as p-values or false discovery rates to assess the significance of the observed differences in metabolite abundance. Additionally, the absence of pathway enrichment analysis for the identified metabolites limits the interpretation of their biological relevance. Integrating this information would enhance the understanding of the metabolic pathways driving the observed changes. Furthermore, providing biological replicates for each stage would increase the robustness of the findings and allow for a more reliable assessment of the observed variations. While the study describes the number of differentially expressed metabolites, further details on their biochemical roles and potential implications in the context of flower development could be included to offer a more comprehensive interpretation. Lastly, considering the complexity of metabolomic data, the inclusion of a validation step, such as targeted quantitative assays for specific metabolites, would strengthen the reliability of the results. Addressing these aspects would contribute to a more thorough and convincing interpretation of the metabolomic dynamics in *Adonis amurensis* flower development.

Autor’s response: We are thankful to the reviewer his/her deep insights. We have revised the section provides information regarding the identification of differentially expressed metabolites, the statistical analysis standards are provided.

We have provided the pathway enrichment analysis for each group comparison, please refer to the (B) part in Figures 6-14. We have used three biological replicates to each stage to increase the robustness of the findings that provide more reliable assessment of the observed variations. Detailed analysis of differentially expressed metabolites between the developmental stages provided insights into their biochemical roles. This has been discussed in the “Discussion” section. To validate our metabolomic data, targeted quantitative assays were performed for specific metabolites. This validation step confirmed the reliability of our findings and provided additional confidence in the observed metabolic shifts. A cluster heatmap (Fig. 2) illustrated the relationships among the different sample groups, with distinct metabolite compositions observed at each developmental stage. Principal Component Analysis (PCA) further highlighted the metabolic dynamics, showing clear separations between non-reproductive (FP, SE, PE) and reproductive (ST, PI) stages. The PCA plot (Fig. 5) indicated significant variance in metabolite expression, particularly during the development of reproductive organs.

5. Discussion
   The discussion in this study, while informative, exhibits certain deficiencies that warrant attention. Firstly, there is a notable absence of a comprehensive exploration of potential limitations or challenges encountered during the research. Information on any technical constraints or methodological considerations in the UPLC-MS/MS analysis is crucial for a thorough evaluation of the study robustness. Additionally, the discussion lacks a detailed analysis of the significance of specific metabolites identified, particularly in relation to their functional roles in flower development. A more in-depth examination of the enriched KEGG pathways, especially how these pathways interconnect and contribute to overall flower development, would enhance the academic value of the study. Furthermore, the PCA plot, while indicating differences in metabolite expression, lacks a thorough interpretation of the ecological or physiological implications of these variances, especially in the context of known physiological processes during flower development. Lastly, the discussion would benefit from suggestions for future research directions or experiments to address the identified gaps in knowledge, thus contributing to the advancement of the field.

Author’s response: We appreciate the reviewer's thoughtful comments and have revised the discussion to address the noted deficiencies. We included a section on potential limitations and challenges, discussing the sensitivity and resolution constraints of the UPLC-MS/MS analysis, potential biases in metabolite detection, sample size considerations, and assumptions made during data normalization and analysis. We expanded our analysis of key metabolites, specifically highlighting the roles of flavonoids such as quercetin and kaempferol, and amino acids such as valine, leucine, and isoleucine in flower development, including their biological significance in reproductive organ development, pollinator attraction, UV protection, and protein synthesis. An in-depth examination of enriched KEGG pathways was added, explaining how pathways like flavonoid biosynthesis and plant hormone signal transduction interconnect and contribute to flower maturation. We provided a thorough interpretation of the PCA plot findings, discussing ecological and physiological implications, particularly during the development of reproductive organs, and relating these findings to specific physiological processes such as pollinator attraction, UV protection, and seed maturation. Additionally, we have provided a section that provides detailed suggestions for future research directions, proposing the expansion of sample size, targeted quantitative assays for specific metabolites, and integrating metabolomic data with transcriptomic and proteomic analyses. We also suggested exploring the anti-cancer and antibacterial potential of specific metabolites and generating transgenic *A. amurensis* plants to study the genetic basis of metabolite production.

6. Conclusion
   The conclusion of the study provides a summary of the systematic investigation into primary and secondary metabolite changes during the developmental stages of *Adonis amurensis* flowers. However, several deficiencies can be identified. Firstly, the conclusion lacks specificity in discussing the nature of the observed changes in primary and secondary metabolites, leaving readers without a detailed understanding of the molecular alterations. Additionally, while the conclusion mentions the association between flower development and metabolite changes, it falls short in providing a comprehensive analysis of the implications of these associations. The statement about the analysis of molecules from other parts showing various metabolic activities during flower development lacks clarity and would benefit from specific examples or details. The suggestion that a specific bioactive compound can be isolated at a particular developmental stage lacks supporting evidence or elaboration on the identified compound. In terms of future prospects, while the study suggests targeting specific metabolite expression for anti-cancer and antibacterial studies, it lacks a concrete plan or methodology for the proposed future research. Furthermore, the mention of generating transgenic plants lacks specificity regarding the genes of interest or the potential challenges associated with this genetic manipulation. Overall, the conclusion could be strengthened by providing more nuanced insights, supporting statements with specific findings, and offering a more detailed roadmap for future research directions.

Author’s response: We have extensively revised the “Conclusion” section by focusing the reviewer’s comments.

In my overall assessment, it is my considered opinion that the manuscript would greatly benefit from revisions in accordance with the insightful suggestions provided. I believe that the proposed changes would contribute significantly to the refinement and enhancement of the scholarly quality of the paper. However, I would like to reserve my final decision until after reviewing the revisions. I appreciate your attention to these suggestions and look forward to examining the refined manuscript.

Author’s response:

We have revised the manuscript considering these comments and provided an updated version. We used the track change option in the word document to record all these changes. Two copies of the manuscript are provided: one clean and one with tracked changes where all revisions are visible. These revisions significantly strengthen the manuscript and enhance its readability. We hope the reviewer finds these improvements satisfactory, enhancing the overall quality and impact of our manuscript. We are more than happy to answer any questions the reviewers may have.
