# Supplementary material for: Metabolite changes during developmental transitions in Adonis amurensis Regel et Radde flowers: Insights from HPLC-MS analysis
Source: PLoS One. 2025 Jan 6;20(1):e0313337. doi: 10.1371/journal.pone.0313337 (PMC11703111; doi:10.1371/journal.pone.0313337)
Supplement: Attachment — Submitted filename: Response to Reviewers.docx [file pone.0313337.s006.docx]

Response letter: PONE-D-23-24707R2

Metabolite changes during developmental transitions in *Adonis amurensis* Regel et Radde flowers: Insights from HPLC-MS analysis

Dear editor,

We are thankful to you and to the reviewers for deeply evaluating our manuscript. We have gone through the new comments carefully and incorporated all of these changes to manuscript.

We have uploaded the following files to the online system.

- A marked-up copy of the manuscript that highlights changes made to the original version. This file is labeled as 'Revised Manuscript with Track Changes'.
- An unmarked version of our revised paper without tracked changes. This file is labeled as 'Manuscript'.

**Journal Requirements:**

Please review your reference list to ensure that it is complete and correct. If you have cited papers that have been retracted, please include the rationale for doing so in the manuscript text, or remove these references and replace them with relevant current references. Any changes to the reference list should be mentioned in the rebuttal letter that accompanies your revised manuscript. If you need to cite a retracted article, indicate the article’s retracted status in the References list and also include a citation and full reference for the retraction notice.

**Response:**

We have double checked all the references and revised them where necessary. All the new changes are visible in the 'Revised Manuscript with Track Changes' version.

Reviewers' comments:

**Comments to the Author**
Reviewer #1: I am pleased to convey that the authors of the major revision have made substantial enhancements to the manuscript, diligently addressing both content and linguistic concerns. Their thorough and thoughtful revisions have significantly improved the overall quality of the manuscript.
After a meticulous review of the revised manuscript, I am confident in stating that the authors have demonstrated a commendable commitment to refining their work. The clarity of expression and the resolution of linguistic issues reflect a high level of professionalism and scholarly dedication.
In light of the authors' careful and comprehensive revisions, I am pleased to affirm that the manuscript is now well-suited for publication. I commend the authors for their diligence and commendable efforts in ensuring the scholarly merit of their work.
Please consider this assessment as a positive recommendation for the publication of the manuscript. I believe that the contributions made by the authors will greatly benefit the academic community, and the manuscript is now poised to make a valuable contribution to the relevant field.

**Response:**

We are thankful to the reviewer for his/her nice words. We will continue our efforts to contribute to scientific community.

Reviewer #4: I have following observations in manuscript that may be incorporated in manuscript:

1. Voucher specimen number/authentification number of A. amurensis needs to be incorporated in material and methods section.

**Response:**

We have added the voucher specimen number to the materials and methods section.

2. What are the different marker metabolites were analysed in present study?

**Response:**

We appreciate the reviewer's insightful question regarding the marker metabolites analyzed in this study. To provide a thorough and detailed answer, we have added a new sub-section titled "Key Findings in Metabolites Across Developmental Stages" before the "Discussion" section in the manuscript. This section comprehensively describes the significant metabolites identified across the different developmental stages of *Adonis amurensis* flowers and highlights their biological relevance.

By presenting this new sub-section in the manuscript, we aim to provide a clearer and more detailed understanding of the complex interactions between these key metabolites and their roles in facilitating flower development in A. amurensis. We believe this addition helps address the reviewer's comment comprehensively, and we are happy to provide further clarifications if needed.

3. Natural compounds generally found in plants in different forms of isomer. How you identified these metabolites on the basis of HPLC-mass analysis. Whether used any reference standard for your study?

**Response:**

We appreciate the reviewer's question about the identification of metabolites, especially regarding the presence of isomers, and the use of reference standards. The identification and accurate annotation of metabolites, particularly those that can exist in different isomeric forms, was a critical aspect of our study. Below, we provide a detailed explanation of our approach:

**Metabolite identification using HPLC-MS/MS:**

The identification of metabolites was performed using High-Performance Liquid Chromatography coupled with Tandem Mass Spectrometry (HPLC-MS/MS). Our analysis utilized Ultra-Performance Liquid Chromatography (UPLC) systems combined with a QTRAP mass spectrometer. This combination allowed us to achieve high sensitivity and resolution, which is essential for distinguishing compounds that may exist as isomers. The system operated under both positive and negative ion modes to maximize the detection of a wide range of metabolites. This method has been widely used in metabolomic studies for its precision and ability to handle complex mixtures. A group of researchers have performed a much similar study to evaluate the metabolome in *Gloriosa superba* ‘Rothschildiana’ during different flower development stages (Sun, Hu et al. 2023).

**Addressing isomeric compounds:**

We recognize that natural compounds can exist in different forms of isomers, which may present challenges during analysis. To address this, we relied on the detailed fragmentation patterns obtained from MS/MS spectra. Each compound's fragmentation pattern (MS/MS spectrum) provides a unique "fingerprint" that helps differentiate isomers, even if they have similar or identical precursor masses. By comparing these fragmentation patterns to known reference spectra, we could accurately identify and distinguish between isomers (Sun, Hu et al. 2023).

**Use of the Metware Database (MWDB):**

For the characterization of substances, we utilized the Metware Database (MWDB), which is a comprehensive metabolomics database containing extensive information on various metabolites, including their spectral data. This database has been validated and extensively used by the research community (Xu, Yan et al. 2020, Zheng, Hao et al. 2021, Liu, Han et al. 2023). The comparison of the acquired MS/MS spectra with the reference spectra in the MWDB allowed us to ensure high accuracy in metabolite identification and annotation, including the differentiation of isomeric compounds.

**Reference standards:**

For the characterization of substances, we utilized the Metware Database (MWDB), which is a comprehensive metabolomics database containing extensive information on various metabolites, including their spectral data. This database has been validated and extensively used by the research community (Zhou, Wang et al. 2020, Xiao, Cao et al. 2021, Zheng, Hao et al. 2021, Xu, Huang et al. 2024). The comparison of the acquired MS/MS spectra with the reference spectra in the MWDB allowed us to ensure high accuracy in metabolite identification and annotation, including the differentiation of isomeric compounds. The reliance on a well-established database like MWDB provided a robust means of verifying the accuracy of our metabolite identification, ensuring consistency and reliability across our findings.

**Quality Control and Data Reproducibility:**

To ensure the reliability and reproducibility of our data, quality control (QC) samples were included throughout the analysis. These QC samples were prepared by pooling equal aliquots from each biological replicate. They were analyzed periodically during the HPLC-MS/MS runs to monitor the stability of the system and the consistency of the data. The low coefficient of variation (CV) values across the QC samples indicated excellent data stability, further reinforcing the reliability of our metabolite identification process (Sands, Gómez-Romero et al. 2021).

We hope this explanation clarifies our methodology for identifying metabolites, particularly those in isomeric forms, and the role of reference standards in our study. Should there be a need for additional information or clarification, we are happy to provide further details.

**References:**

Liu, J., L. Han, G. Li, A. Zhang, X. Liu and M. Zhao (2023). "Transcriptome and metabolome profiling of the medicinal plant Veratrum mengtzeanum reveal key components of the alkaloid biosynthesis." Frontiers in Genetics **14**.

Sands, C. J., M. Gómez-Romero, G. Correia, E. Chekmeneva, S. Camuzeaux, C. Izzi-Engbeaya, W. S. Dhillo, Z. Takats and M. R. Lewis (2021). "Representing the Metabolome with High Fidelity: Range and Response as Quality Control Factors in LC-MS-Based Global Profiling." Analytical Chemistry **93**(4): 1924-1933.

Sun, Y., P. Hu, Y. Jiang, J. Li, J. Chang, H. Zhang, H. Shao and Y. Zhou (2023). "Integrated Metabolome and Transcriptome Analysis of Petal Anthocyanin Accumulation Mechanism in Gloriosa superba 'Rothschildiana' during Different Flower Development Stages." Int J Mol Sci **24**(20).

Xiao, L., S. Cao, X. Shang, X. Xie, W. Zeng, L. Lu, Q. Kong and H. Yan (2021). "Metabolomic and transcriptomic profiling reveals distinct nutritional properties of cassavas with different flesh colors." Food Chemistry: Molecular Sciences **2**: 100016.

Xu, J., J. Yan, W. Li, Q. Wang, C. Wang, J. Guo, D. Geng, Q. Guan and F. Ma (2020). "Integrative analyses of widely targeted metabolic profiling and transcriptome data reveals molecular insight into metabolomic variations during apple (Malus domestica) fruit development and ripening." International Journal of Molecular Sciences **21**(13): 4797.

Xu, W., J. Huang, P. Wang, Y. Yang, S. Fu, Z. Ying and Z. Zhou (2024). "Using widely targeted metabolomics profiling to explore differences in constituents of three Bletilla species." Scientific Reports **14**(1): 23873.

Zheng, S., Y. Hao, S. Fan, J. Cai, W. Chen, X. Li and X. Zhu (2021). "Metabolomic and transcriptomic profiling provide novel insights into fruit ripening and ripening disorder caused by 1-MCP treatments in papaya." International Journal of Molecular Sciences **22**(2): 916.

Zhou, Y., Z. Wang, Y. Li, Z. Li, H. Liu and W. Zhou (2020). "Metabolite profiling of sorghum seeds of different colors from different sweet sorghum cultivars using a widely targeted metabolomics approach." International journal of genomics **2020**(1): 6247429.
